# Keratin 17 Is Induced in Oral Cancer and Facilitates Tumor Growth

**DOI:** 10.1371/journal.pone.0161163

**Published:** 2016-08-11

**Authors:** Rumana Khanom, Chi Thi Kim Nguyen, Kou Kayamori, Xin Zhao, Keiichi Morita, Yoshio Miki, Ken-ichi Katsube, Akira Yamaguchi, Kei Sakamoto

**Affiliations:** 1 Department of Oral Pathology, Graduate School of Medical and Dental Sciences, Tokyo Medical and Dental University, Tokyo, Japan; 2 Department of Oral and Maxillofacial Surgery, Graduate School of Medical and Dental Sciences, Tokyo Medical and Dental University, Tokyo, Japan; 3 Department of Molecular Genetics, Medical Research Institute, Tokyo Medical and Dental University, Tokyo, Japan; 4 Department of Nursing Science, Faculty of Human Care, Tohto College of Health Sciences, Saitama, Japan; 5 Oral Health Science Center, Tokyo Dental College, Tokyo, Japan; 6 Global Center of Excellence (GCOE) Program, “International Research Center for Molecular Science in Tooth and Bone Disease”, Tokyo Medical and Dental University, Tokyo, Japan; Universitat Wien, AUSTRIA

## Abstract

Keratin subtypes are selectively expressed depending on the cell type. They not only provide structural support, but regulate the metabolic processes and signaling pathways that control the growth of the epithelium. KRT17 (keratin 17) is induced in the regenerative epithelium and acts on diverse signaling pathways. Here, we demonstrate that KRT17 is invariably and permanently induced in oral squamous cell carcinoma (OSCC), as revealed by immunohistochemistry and cDNA microarray analysis. Two representative OSCC cell lines; KRT17-weakly expressing Ca9-22 and KRT17-highly expressing HSC3 were used to establish KRT17-overexpressing Ca9-22 and KRT17-knockdown HSC3 cells. Analysis of these cells revealed that KRT17 promoted cell proliferation and migration by stimulating the Akt/mTOR pathway. KRT17 also upregulated the expression of SLC2A1 (solute carrier family 2 member 1/Glut1) and glucose uptake. To further investigate the effect of KRT17 on tumorigenesis, KRT17-knockout HSC3 cells were established and were transplanted to the cephalic skin of nude mice. The tumors that developed from KRT17-knockout HSC3 cells had a lower Ki-67 labeling index and were significantly smaller compared to the controls. These results indicate that KRT17 stimulates the Akt/mTOR pathway and glucose uptake, thereby facilitating tumor growth. We could not confirm the relationship between KRT17 and SFN (stratifin) in the cells examined in this study. However, our study reinforces the concept that the cellular properties of cancer are regulated by a series of molecules similar to those found in wound healing. In OSCC, KRT17 acts as a pathogenic keratin that facilitates tumor growth through the stimulation of multiple signaling pathways, highlighting the importance of KRT17 as a multifunctional promoter of tumorigenesis.

## Introduction

Keratins are a family of epithelial-specific intermediate filament proteins, and the KRT gene family is the largest in humans, with 54 functional genes. Keratins can be classified as type I or type II and are arranged in heterotypic pairs [1]. Their expression is highly cell type specific, making them excellent markers for specific lineage and differentiation [2, 3]. Keratins provide structural support, regulate metabolic processes, and stimulate intracellular signaling pathways that regulate the growth of epithelium [4]. In the non-cornified epithelium of oral, esophageal, and vaginal mucosae, keratin 4 (KRT4) and KRT13 are expressed in the suprabasal layer and KRT5, KRT14, KRT15, and KRT19 are expressed in the basal layer. These normal expression patterns of keratins are altered under various physiological and pathological conditions.

In injured skin, KRT6, KRT16, and KRT17 are rapidly induced in the epidermis at the wound margin [5–8]. These regeneration-related keratins give rise to phenotypic changes in the epithelium. In injured skin of *Krt6* knockout mice, the epidermis undergoes lytic degeneration and becomes fragile, suggesting that KRT6 gives tensile strength to the regenerative epithelium [9]. The primary function of keratins is mechanical stabilization of cell shape, but accumulating evidence suggests that they also perform non-mechanical functions of modulating signaling pathways. *Krt16* transgenic mice showed activation of EGF signaling, which resulted in epidermal hypertrophy due to increased cell proliferation, indicating that KRT16 makes epithelial cells more sensitive to signaling cues in regeneration [10]. Cancer cells often exhibit abnormal keratin expression [3]. We previously engaged in comprehensive keratin profiling in oral squamous cell carcinoma (OSCC) and found that KRT6, KRT16, and KRT17 were upregulated [11]. In particular, induction of KRT17 was observed most clearly, prompting us to further explore its relevance in the pathogenesis of OSCC.

KRT17 influences the keratinocyte behaviors in cutaneous wound healing; *Krt17* knockout mice manifest a delay in wound healing [12]. This is caused by reduced activities of AKT1 and MTOR (mammalian target of rapamycin), suggesting that KRT17 substantiates cell growth by promoting protein synthesis. This signaling activity of KRT17 is seemingly accomplished through interaction with the multifunctional adaptor protein SFN (stratifin/14-3-3-σ). KRT17 binds to SFN and recruits it to the cytoplasm, where SFN stimulates the Akt/mTOR pathway [12, 13]. A unique contribution of keratin to the mTOR pathway has been suggested by another line of evidence. Vijayaraj et al. generated mice that lacked the entire type II keratin gene cluster [14]. These keratin-null mice displayed severe growth retardation due to defective glucose uptake. This was attributed to mislocalization of the glucose transporters SLC2A1 (solute carrier family 2 member 1/Glut1) and SLC2A3 in the yolk sac epithelium, which falsely suppressed the mTOR signaling [14]. Squamous cell carcinoma and cutaneous wounds are different pathological states; however, they share many common features, such as activation of epithelial cells, induction of regeneration-related keratins, stimulation of cell growth and migration, and remodeling of connective tissue. We hypothesized that cellular properties of OSCC are regulated by molecular mechanisms similar to those in the regenerative epithelium [15–17], and KRT17 may play a substantial role in the regulation of cancer cell behaviors.

In this study, we show that KRT17 is invariably induced in OSCC and stimulates the Akt/mTOR pathway and SLC2A1 expression, thereby facilitating tumor growth. Our findings indicate the importance of KRT17 as a tumorigenic factor in OSCC.

## Materials and Methods

### Immunohistochemistry and cDNA microarray analysis

Fifty specimens of OSCC and 10 specimens of epulis with ulcer containing regenerative epithelium were collected from the archives of the Dental Hospital of Tokyo Medical and Dental University. A tissue microarray was constructed as previously described [18]. The experimental procedures were approved by the ethics committee of Tokyo Medical and Dental University (Registration No. 99 and No. 991). Since the archival tissue specimens were obtained for pathology diagnosis, the ethics committee approved waiver of specific informed consent in accordance with Ethical Guidelines for Clinical Studies by Ministry of Health, Labor and Welfare of Japan. Immunohistochemical staining was conducted as previously described [19]. cDNA microarray analysis of OSCC cells was performed as previously described [20]. Written informed consents were obtained from the participants of the cDNA microarray analysis.

### Antibodies

Primary antibodies used in this study were anti-KRT17 (D7327, Cell Signaling Technology, Danvers, MA, USA), KRT17 (EPR1624Y, Abcam, Cambridge, UK), KRT17 (E2, Dako, Glostrup, Denmark), KRT5 (EP1601Y, Abcam), KRT6 (EPR1603Y, Abcam), KRT16 (EP1615Y Abcam), mTOR (Y391, Abcam), mTOR (7C10, Cell Signaling Technology), 4E-BP1 (53H11, Cell Signaling Technology), phospho-4E-BP1(Thr37/46)(236B4, Cell Signaling Technology), AKT1 (Y89, Abcam), AKT1 (phospho S473)(EP2109Y, Abcam), GAPDH (D16H11, Cell Signaling Technology), Histone H3 (D1H2, Cell Signaling Technology), Beta-Tubulin (9F3, Cell Signaling Technology), 14-3-3 sigma (1.N.6, Abcam), Glut1 (EPR3915, Abcam), Ki67 (SP6, Abcam), Digoxigenin (Roche Diagnostics, Basel, Switzerland), Flag M2 (Sigma-Aldrich, St. Louis, MO, USA), HA (12CA5, Roche Diagnostics) antibodies. Secondary antibodies used in this study were Envision/HRP (Dako), HRP-donkey anti-rabbit IgG (Thermo Fisher Scientific, Waltham, MA, USA), HRP-rabbit anti-mouse IgG (Thermo Fisher Scientific), Alexa Fluor 488 goat anti-rabbit IgG (Thermo Fisher Scientific), Alexa Fluor 594 goat anti-rabbit IgG (Thermo Fisher Scientific) and Alexa Fluor 488 rabbit anti-mouse IgG (Thermo Fisher Scientific).

### Cell culture

Ca9-22, HSC5 and 293T cell lines were obtained from the RIKEN Bioresource Center (Tsukuba, Japan). HSC3 and HO-1-N-1 were obtained from the Japanese Collection of Research Bioresources (Osaka, Japan). BHY was kindly provided by Dr. Masato Okamoto. HSC4 was kindly provided by Dr. Masao Saito. The cells were maintained in Dulbecco’s modified Eagle’s medium with 10% fetal bovine serum. Primary human foreskin keratinocytes were obtained from Kurabo (Osaka, Japan).

### Immunocytochemistry

Cells were fixed in methanol for 5 min, rinsed 3 times with PBS, and incubated in primary antibody (1:500 dilution) solution at room temperature for 2 h. The cells were rinsed 3 times with PBS and were incubated in fluorescence-conjugated secondary antibody solution at room temperature for 1 h. After rinsing 3 times with PBS, the cells were mounted in mounting medium.

### RT-PCR, northern blot, and western blot analysis

Extraction of RNA from cells was performed using the NucleoSpin RNA II (Macherey-Nagel, Düren, Germany). RNA was reverse transcribed into cDNA using a mixture of oligo(dT) and random primers. PCR was performed using the PrimeSTAR GXL DNA Polymerase (Takara, Shiga, Japan). PCR primer sequences were as follows. *KRT17*; *CCCACTTGGTGGCCTATAAA* and *GTCATCAGGCAAGGAAGCAT*. *GAPDH*; *GCACCGTCAAGGCTGAGAAC* and *ATGGTGGTGAAGACGCCAGT*. Northern blot analysis was conducted using an RNA probe made with the Dig RNA labeling Mixture (Roche Diagnostics) following the manufactures instructions. Protein extraction from cells was carried out using RIPA or NP40 lysis buffer. Protein extraction from formalin-fixed paraffin-embedded specimen was performed as previously described using the protein extraction buffer (50 mM Tris (pH = 8.0), 5 mM EDTA, 2% SDS) [21]. Separate extraction of cytoplasmic and nuclear proteins was performed using the DUALXtract (Dualsystems Biotech, Zurich, Switzerland). Western blot analysis was conducted as previously described (Current Protocols in Molecular Biology, ISBN: 9780471142720). Densitometric analysis of proteins on SDS-PAGE gels was performed using the NIS-Elements Basic Research (Nikon, Tokyo, Japan). Expression level of protein X in a clone was normalized against beta1-tubulin (TUBB) and then against the control cells using the formula (Value of protein X in the clone / Value of TUBB in the clone) / (Value of protein X in the control cells / Value of TUBB in the control cells). The normalized expression levels were plotted on a log scale.

### Generation of KRT17 overexpressing and knockdown cells

Human *KRT17* cDNA was obtained by RT-PCR using RNA taken from the gingiva of a volunteer. The primer sequences were *CCCACTTGGTGGCCTATAAA* and *GTCATCAGGCAAGGAAGCAT*. The *KRT17* cDNA was cloned into *pcDNA3* (Thermo Fisher Scientific). *KRT13* cDNA was obtained as previously described [11]. The cells were transfected with the plasmid using FuGene6 (Roche Diagnostics), and G418 (500 μg/ml)-resistant clones were isolated. Knockdown plasmid was generated using the BLOCK-iT Pol II miR RNAi Expression Vector Kits (Thermo Fisher Scientific). Transfection was carried out using the Calcium Phosphate Transfection Kit (Thermo Fisher Scientific) and the cells were selected in blasticidin (5 μg/ml).

### Proliferation, migration, invasion and glucose uptake assays

Cell proliferation rate was evaluated by measuring the metabolic activity using the Cell Counting Kit-8 (Dojindo Molecular Technologies, Tokyo, Japan). Apoptosis was assayed using the Alexa Fluor 488 Annexin V/Dead Cell Apoptosis Kit (Thermo Fisher Scientific). Migration assay was conducted using ThinCert of 8 μm pores (Greiner Bio-One, Wemmel, Belgium). Cells (2x10^5^ cells/L) were suspended in serum-free medium and seeded into the upper chamber. The lower chamber was filled with serum-containing medium. Forty-eight hours later, cells that migrated into the lower chamber through the filter were stained by crystal violet and manually counted in 3 microscopic fields per sample. Proliferation inhibitors were not used in the migration and invasion assays. For invasion assay, the ThinCert cell culture inserts were coated by BD matrigel (BD Biosciences, San Jose, CA, USA) diluted at 1:50 in DMEM. Rapamycin was purchased from Wako Pure Chemical Industries (Osaka, Japan) and perifosine was purchased from LC Laboratories (Woburnm, MA, USA). Glucose uptake was measured using the Glucose uptake assay kit (Cayman Chemical Company, Ann Arbor, MI, USA) following the manufacturer’s instructions. The cells were treated with the fluorescence glucose analogue for 2 h. The fluorescence image was digitally analyzed using the NIS-Elements Basic Research (Nikon, Tokyo, Japan) and the signals in individual cells were depicted as brightness units.

### Generation of KRT17 knockout cells

The knockout plasmid that targets the exon 2 of *KRT17* was constructed using *pSpCas9(BB)-2A-GFP* (Addgene plasmid 48138). The guide sequence was chosen using the CRISPR design tool [22]. The guide sequence was *CGGCCTGGGCAGCACCCTCG* that corresponded from +259 to +278 of *KRT17* cDNA when the A of the ATG of the initiator methionine codon was designated as position +1. HSC3 cells were transfected with the plasmid using Polyethylenimine Max (Polysciences, Warrington, PA, USA) and GFP-positive cells were sorted 48 h after transfection using FACS Aria II (BD Biosciences) and cloned by limiting dilution.

### Xenograft of human cancer cells in athymic mice

All mice were housed in groups of four in plastic cages in a temperature-controlled room with proper dark-light cycles, and were provided with tap water and food *ad libitum*. Cells (5 x 10^5^) were subcutaneously injected into the cephalic skin of athymic mice (BALB/c Slc-nu/nu), as previously described [23]. The health of the animals was monitored twice per day. Behavioral changes such as reduced exploration were used as criteria to determine a humane endpoint. None of the mice reached the endpoint and they all exhibited active exploratory behavior until the day of sacrifice. The animals were euthanized by cervical dislocation. The animal studies were reviewed and approved by the institutional animal care and use committee of Tokyo Medical and Dental University (Registration No. 0150213A).

### Immunoprecipitation

For immunoprecipitation experiments, *KRT13* and *KRT17* were N-terminally HA-tagged by ligating the PCR products into *HA-pcDNA3* (personal product). *SFN* was obtained from Addgene (Addgene plasmid 12453) [22, 24]. 293T cells were transfected with combination of plasmids and the cells were lysed in NP40 lysis buffer 48 h after transfection. Immunoprecipitation was conducted using anti-Flag M2 affinity gel (Sigma-Aldrich).

### Statistical analysis

Correlation between the mRNA expression scores was assessed using Pearson product-moment correlation coefficients. The statistical significance of the r coefficient was examined using the Student’s t-test. The correlations between the expression score and the differentiation grade or the presence of lymph node metastasis were evaluated using the Kruskal–Wallis test or the Mann–Whitney U-test, respectively. The results of proliferation, migration and invasion assays were analyzed using the Student’s t-test. The results of the glucose uptake assay measured by brightness unit were analyzed using the Mann–Whitney U-test. The results of glucose uptake assay measured by flow cytometry were analyzed using the Student’s t-test. The tumor size and Ki67 labeling index were analyzed using the Mann–Whitney U-test. A P-value < 0.05 was considered statistically significant; a P-value < 0.1 was considered to represent a marginally significant tendency.

## Results

### KRT17 is induced in the regenerative epithelium of the oral mucosa

**Fig 1:**
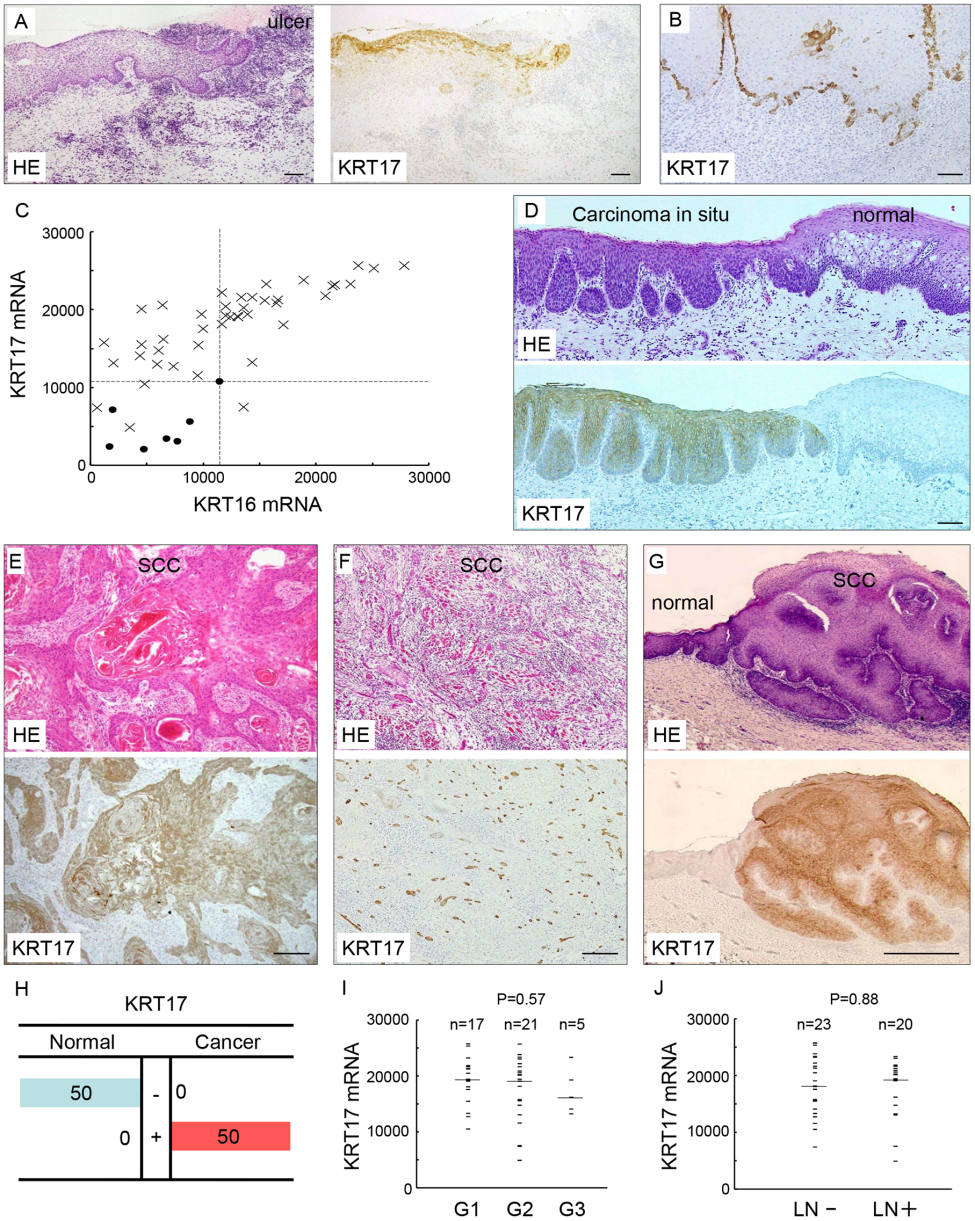
KRT17 expression was induced in the regenerative epithelium and oral squamous cell carcinoma (OSCC). (A) KRT17 expression in regenerative epithelium. Hematoxylin and eosin (HE) staining (left panel) and immunohistochemical staining using the anti-KRT17 antibody (right panel). KRT17 expression was induced in the regenerative epithelium at the edge of the traumatic ulcer of the oral mucosa. Scale bar, 100 μm. (B) KRT17 expression in hyperplastic epithelium. Immunohistochemical staining using the anti-KRT17 antibody. In this buccal mucosa with nonspecific inflammation, KRT17 expression was induced in the basal cells. Note the scattered distribution of KRT17-positive cells in the basal layer. Scale bar, 100 μm. (C) cDNA microarray analysis of *KRT16* and *KRT17* in 43 OSCCs and 7 normal oral epithelia. The scales on the horizontal and vertical axes represent absolute signal values. Crosses denote each OSCC case and filled circles denote normal controls. The dashed lines were placed at the maximum values in the normal epithelium. (D-G) Immunohistochemical expression of KRT17 in OSCC. (D) KRT17 was expressed in the carcinoma *in situ* (left side), whereas KRT17 was not expressed in the normal epithelium (right side). Scale bar, 100 μm. (E, F, G) HE staining (upper panels) and immunohistochemical staining using the anti-KRT17 antibody (lower panels) in OSCC specimens. The upper and lower panels are serial sections from one sample. KRT17 expression was constantly induced in OSCC regardless of the histology. The levels and distributions of expression showed little variation across cases. Scale bar, 100 μm. (E) Keratinizing tumor nests and (F) small tumor islands of OSCC, both showing similar levels of ubiquitous KRT17 expression. Scale bars, 200 μm (E) and 100 μm (F). (G) Condylomatous tumor that showed highly differentiated histology was also positive for KRT17. Normal epithelium was present at the left side, showing negative staining. Scale bar, 1 mm. (H) Summary of immunohistological KRT17 expression in 50 OSCC cases. +, strong staining; -, weak or no staining. (I) Comparison between *KRT17* expression and histological grade of differentiation in 43 OSCCs. G1, well differentiated; G2, moderately differentiated; G3, poorly differentiated. Bars depict the median. N is the total number of cases analyzed. (J) Comparison between the *KRT17* expression and presence of lymph node metastasis in 43 OSCCs. Bars depict the median. N is the total number of cases analyzed.

We first investigated KRT17 expression in the regenerative epithelium of the oral mucosa. Immunohistochemical analysis revealed that KRT17 was induced in all 10 regenerative epithelia examined (Fig 1A), whereas its expression was absent in normal oral mucosa (S1 Fig). The KRT17-positive cells in the regenerative epithelium were sparsely distributed and mixed with KRT17-negative epithelial cells. We also noticed that KRT17 was scattered in the basal layer of the hyperplastic epithelium (Fig 1B). These results indicate that KRT17 is temporally induced when the oral epithelium attains a hyperproliferative state.

### KRT17 is induced in OSCC

Our previous cDNA microarray analysis revealed that *KRT16* and *KRT17* were upregulated significantly in OSCC compared with the normal epithelium (P < 0.001) [11], showing 2.0- and 2.9-fold increases on average, respectively. To examine the correlation of *KRT16* and *KRT17* expression in each case, the data was represented as scatter plots (Fig 1C). For convenience, the plot area was divided into four quadrants according to the maximum values in the normal epithelium (Fig 1C). About two-thirds of the cancers were plotted in the 1^st^ quadrant, which represents concurrent upregulation of *KRT16* and *KRT17*. About one-third of the cancers were plotted in the 2^nd^ quadrant, which represents upregulation of only *KRT17*. Only one cancer was plotted in the 4^th^ quadrant, which represents upregulation of only *KRT16* (Fig 1C). This observation indicates that *KRT17* was expressed more consistently than *KRT16* in OSCC, indicating that KRT17 is a more essential keratin in OSCC. To validate the microarray results, we immunohistochemically examined KRT17 expression in 50 surgically excised OSCC specimens. KRT17 was robustly expressed in OSCC regardless of the histological variations. Representative samples including *in situ* carcinoma (Fig 1D), keratinized cancer nests in well-differentiated cancer (Fig 1E), small cancer nests in poorly differentiated cancer (Fig 1F) and a well-differentiated condylomatous tumor (Fig 1G) demonstrated ubiquitous expression of KRT17 in cancer. We previously reported that among the type II keratins, the expression of KRT5 and KRT6 was increased in OSCC [11, 19]. We confirmed this result from the immunohistochemistry of representative cases (n = 3, S2A Fig) and the upregulation of KRT5 and KRT6 was confirmed by western blot analysis (S2B Fig). The other type II keratins did not show notable expression (data not shown), suggesting that KRT5 and KRT6 act as binding partners of KRT17 in OSCC. In the OSCC cell line HSC3, KRT17 exhibited filamentous localization in the cytoplasm, which was identical to that of KRT5 and KRT6 (S3 Fig). This indicates that KRT17 is incorporated into the cytoskeleton along with KRT5 and KRT6.

The 50 OSCC specimens mentioned above contained normal mucosa, allowing us to compare the expression between OSCC and the normal epithelium in one specimen. Semiquantitative evaluation by immunohistochemical staining demonstrated that KRT17 was significantly upregulated in OSCC compared with the normal oral epithelium (P < 0.001, Fig 1H). The expression in cancer was observed in both the basal and the suprabasal cells, where the suprabasal cells, especially the keratinized cells, tended to be stained more intensely than the basal cells. The mRNA expression in 43 OSCCs did not show significant correlation with the histological grades of differentiation (P = 0.57, Fig 1I) or the presence of lymph node metastasis (P = 0.88, Fig 1J). These results suggest that the induction of KRT17 expression is a common feature of OSCC, and is not associated with case-dependent variable features of OSCC.

It is worth noting the difference in the expression pattern between normal reactive cells and OSCC; expression in the former was dispersed and in the latter it was ubiquitous, revealing a fundamental difference between these cells. The dispersed pattern indicates that the induction of KRT17 in normal cells is a temporary event. The ubiquitous pattern in OSCC underscores the fact that the OSCC cells are permanently converted to express KRT17. These results suggest that KRT17 is an essential factor that characterizes OSCC.

### KRT17 promotes cell proliferation and migration

**Fig 2:**
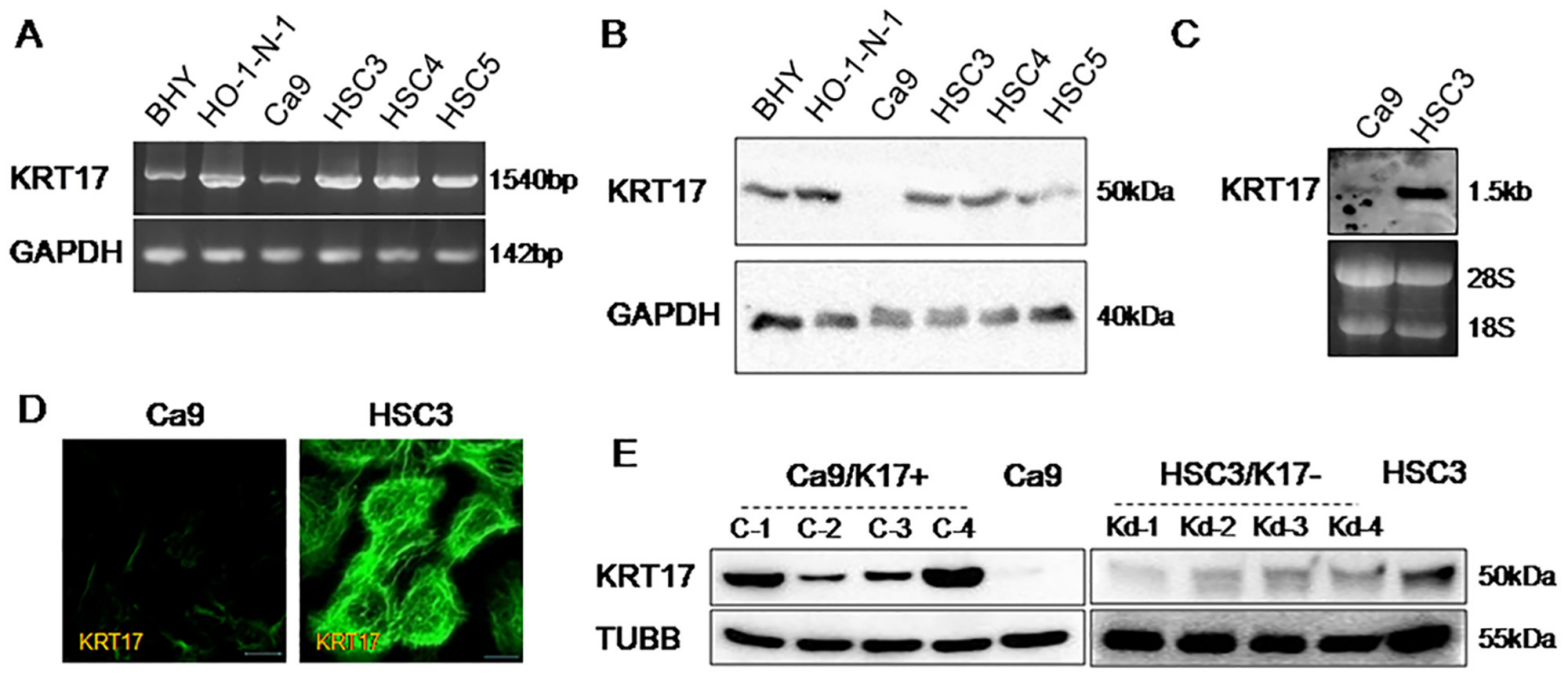
Generation of KRT17-overexpressing and KRT17-knockdown oral cancer cell lines. (A) Agarose gel electrophoresis of RT-PCR assays for the identification of *KRT17* and *GAPDH* in various oral cancer cell lines. (B) Western blot analysis of various oral cancer cell lines for detection of KRT17 and GAPDH. (C) Northern blot analysis of Ca9 and HSC3 cells by hybridization with a *KRT17* probe. (D) Immunofluorescent images showing filamentous staining of KRT17 in Ca9 (faint) and HSC3 cells. Scale bar, 10 μm. (E) Western blot analysis of KRT17-overexpressing Ca9 (Ca9/K17+) clones (C-1, C-2, C-3, and C-4), the parental Ca9 cells, KRT17-knockdown HSC3 (HSC3/K17-) clones (Kd-1, Kd-2, Kd-3, and Kd-4) and the parental HSC3 cells for detection of KRT17 and beta1-tubulin (TUBB). *KRT17* cDNA was transfected into Ca9 to make KRT17-overexpressing cells (Ca9/K17+), and four independent clones (C-1, C-2, C-3, and C-4) were established. KRT17 expression in HSC3 was suppressed by miRNA-mediated knockdown, and four independent clones (Kd-1, Kd-2, Kd-3, and Kd-4) of KRT17-knockdown cells (HSC3/K17-) were established.

In primary human foreskin (HFS) cells, KRT17 expression was strong in subconfluent culture, but the expression diminished after 72 h of confluency (S4 Fig). This result is in line with the histological finding that KRT17 was absent in the normal epithelium and was induced in a hyperproliferative state. Next we examined the expression of KRT17 in oral cancer cell lines. Reverse transcription polymerase chain reaction (RT-PCR) showed that all the oral cancer cell lines examined expressed *KRT17* (Fig 2A). Western blot analysis showed that KRT17 expression was much lower in Ca9-22 (referred to as Ca9 hereafter) compared with the other cells (Fig 2B). KRT5, KRT6 and KRT16 were differentially expressed in these cells (S5 Fig). We chose two representative cell lines: KRT17-highly expressing HSC3 and KRT17-weakly expressing Ca9. HSC3 is a cell line established from tongue squamous cell carcinoma and has high metastatic potential. Ca9 is a cell line established from gingival squamous cell carcinoma; it retains the phenotype of normal keratinocytes relatively well compared with other oral cancer cell lines [21]. Northern blot analysis confirmed that *KRT17* expression was high in HSC3 and low in Ca9 (Fig 2C). Immunocytostaining further confirmed strong KRT17 expression in HSC3 and weak KRT17 expression in Ca9 (Fig 2D). KRT17 was contained in cytoplasmic filaments in both HSC3 and Ca9 (faintly).

**Fig 3:**
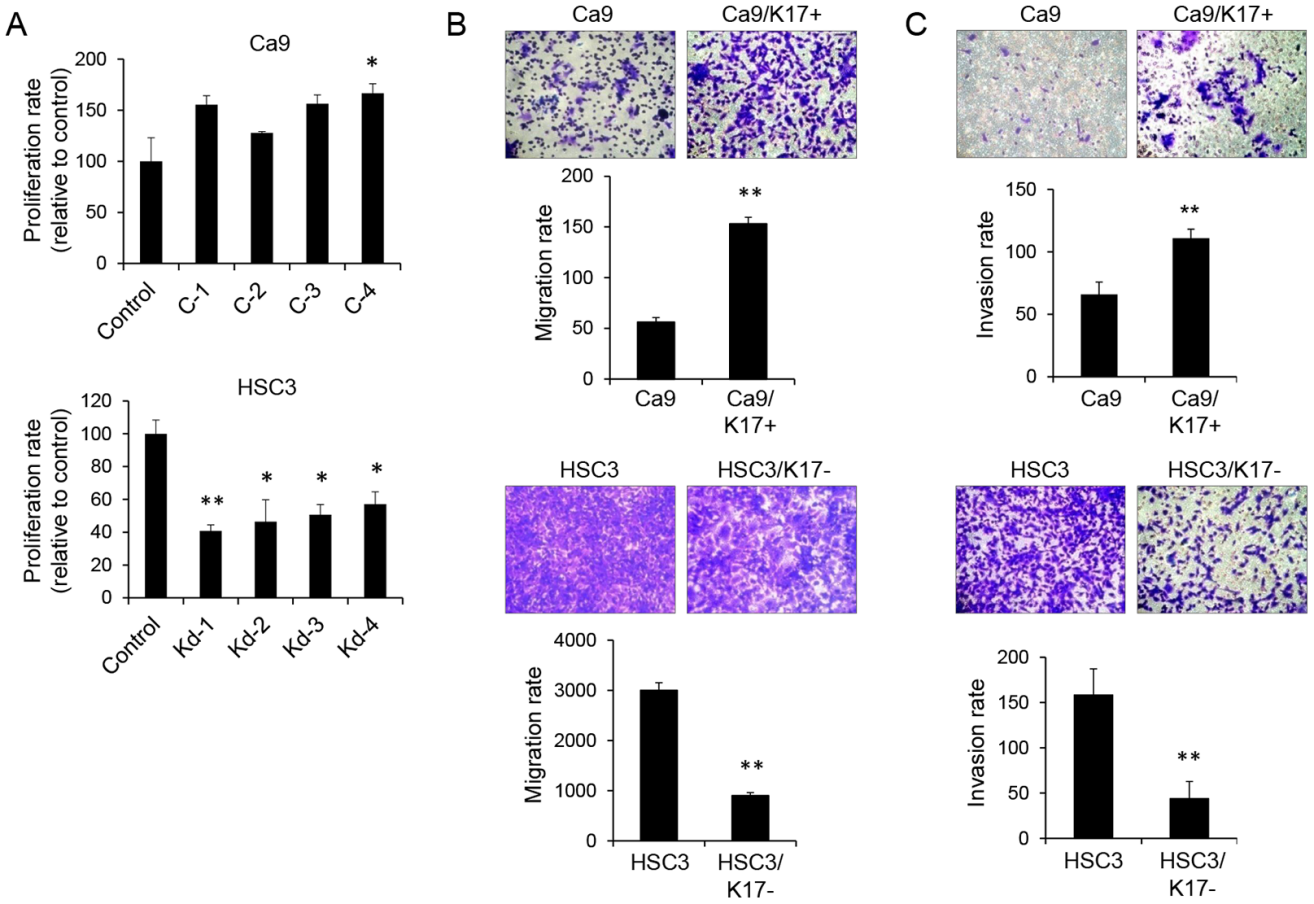
KRT17 promoted cell proliferation and migration. (A) Cell proliferation assay after 72h of culture. C-4 showed a significantly higher rate of proliferation than the control. *P < 0.05 compared with the control. C-1, C-2, and C-3 showed a tendency towards elevated proliferation (0.05 < P < 0.1). HSC3/K17- showed significantly lower rates of proliferation than the control. Representative graphs of n = 3 independent experiments (each experiments comprised three technical replicates). **P < 0.01 and *P < 0.05 compared with the control. Data represent mean ± SEM. (B) Transwell migration assay using Boyden chambers. Cells (2 x 10^5^ cells/L) were suspended in serum-free medium and seeded into the upper chamber with pores of 8 μm. The lower chamber was filled with serum-containing medium. The upper chamber was confluent with cells during the assay. After 48 h, the cells that had migrated into the lower chamber through the filter were stained with crystal violet and counted in three microscopic fields per sample. Representative graphs of n = 3 independent experiments (each experiments comprised three technical replicates). **P < 0.01 compared with the control. Data represent mean ± SEM. (C) Transwell invasion assay. The filters were coated with an extracellular matrix protein mixture, and a transwell migration assay was conducted. Representative graphs of n = 3 independent experiments (each experiment comprised three technical replicates). **P < 0.01 compared with the control. Data represent mean ± SEM.

We established four independent clones of KRT17-overexpressing Ca9 (referred to as Ca9/K17+) and four independent clones of KRT17-knockdown HSC3 (referred to as HSC3/K17-), and examined the effects of KRT17 on their cell properties. Western blot analysis confirmed increased expression of KRT17 in Ca9/K17+ compared with Ca9, and decreased expression of KRT17 in HSC3/K17- compared with HSC3 (Fig 2E). KRT17 in Ca9/K17+ colocalized with KRT5 and KRT6 without showing any aggregation or ectopic accumulation of KRT17 (S6 Fig), indicating that the overexpressed KRT17 was functioning normally in Ca9/K17+. We measured cell proliferation using a metabolic cell proliferation assay. Ca9/K17+ showed higher rates of proliferation than the control (P < 0.05 in C-4; P < 0.1 in C-1, C-2, and C-3). HSC3/K17- showed significantly lower rates of proliferation than the control (P < 0.05; Fig 3A). Apoptosis and cell death were examined by cell staining using fluorescently-labeled anti-annexin V antibody and propidium iodide. Virtually all the cells were negative for annexin V and propidium iodide (data not shown), excluding the influence of cell death on the result of the cell proliferation assay. In the following experiments, we display the results using C-4 and Kd-4 as representatives, but the other clones yielded comparable results. A Boyden chamber assay revealed that Ca9/K17+ showed significantly higher rates of migration than Ca9 (P < 0.01; Fig 3B, upper panel). HSC3/K17- showed significantly lower rates of migration than HSC3 (P < 0.01; Fig 3B, lower panel). An invasion assay using culture inserts coated with basement membrane components revealed that transwell invasion was significantly increased in Ca9/K17+ compared with the control (P < 0.01; Fig 3C, upper panel), whereas it was significantly decreased in HSC3/K17- compared with the control (P < 0.01; Fig 3C, lower panel). These results suggest that KRT17 promotes cell proliferation and migration.

### KRT17 exerts its effects via the Akt/mTOR signaling pathway

**Fig 4:**
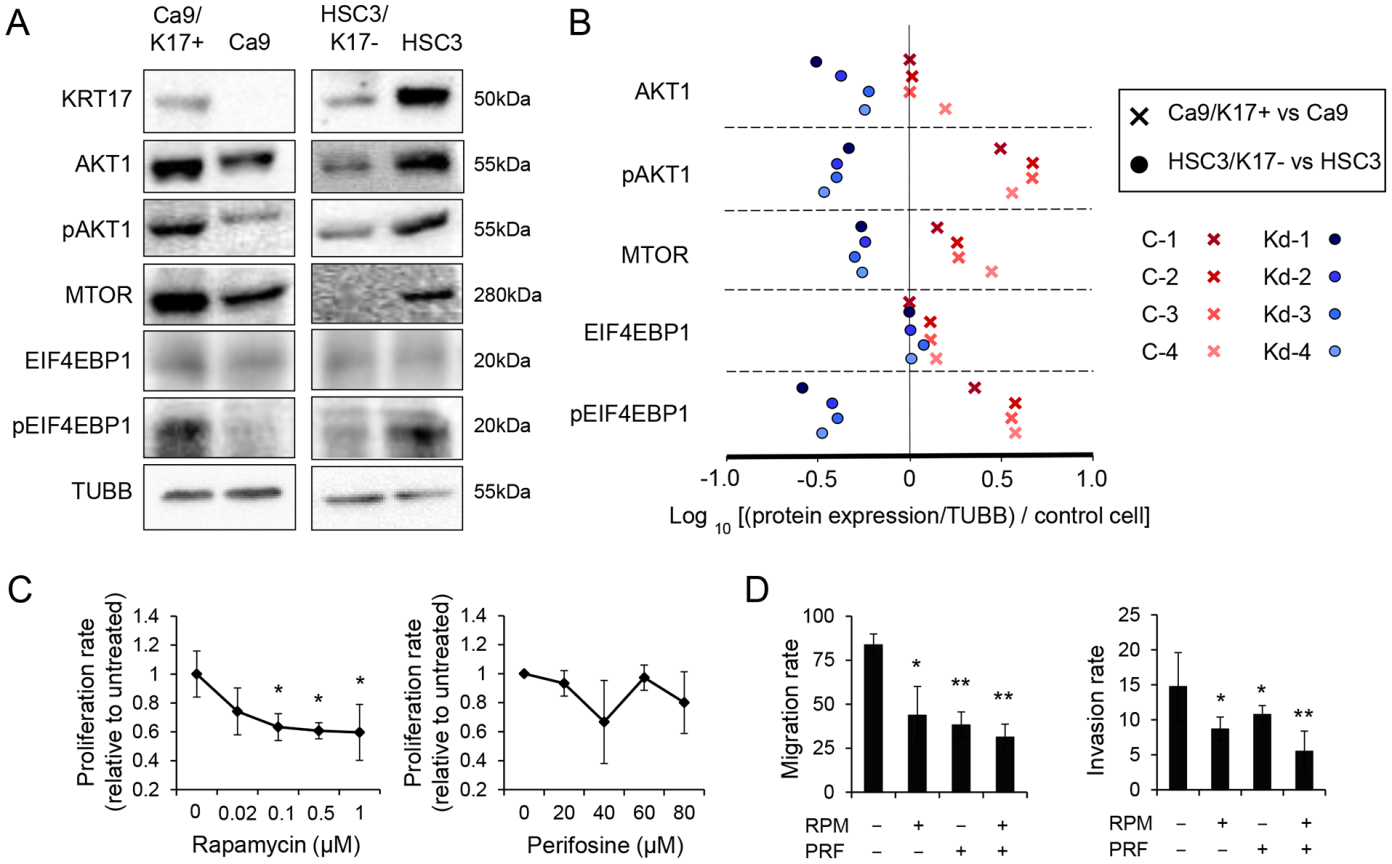
KRT17 stimulated the Akt and mTOR pathway. (A) Representative images of western blot analysis of Ca9/K17+ (C-4), Ca9, HSC3/K17- (Kd-4), and HSC3 for detecting KRT17, AKT1, phosphorylated AKT1 (pAKT1), MTOR, EIF4EBP1, phosphorylated EIF4EBP1 (pEIF4EBP1), and beta1-tubulin (TUBB). Quantitative results measured by densitometric analysis of all the clones (C-1 to C-4 and Kd-1 to Kd-4) are shown in (B). Representative images of two assays. (B) Expression of AKT1, pAKT1, MTOR, EIF4EBP1 and pEIF4EBP1 in Ca9/K17+ (C-1, C-2, C-3, and C-4) and HSC3/K17- (Kd-1, Kd-2, Kd-3, and Kd-4) compared with Ca9 and HSC3, respectively, as revealed by densitometric analysis of the western blots. Expression level of protein X in a clone was normalized against TUBB and then against the control cells using the formula (Value of protein X in the clone / Value of TUBB in the clone) / (Value of protein X in the control cells / Value of TUBB in the control cells). The normalized expression levels were plotted on a log scale. Representative results of two independent assays that showed similar results. (C) Effect of mTOR-inhibitor rapamycin (RPM) and Akt-inhibitor perifosine (PRF) on proliferation of Ca9/K17+ (C-4) cells. Ca9/K17+ cells were treated with different concentrations of either RPM or PRF for 72 h and cell densities were measured. Representative graphs of three assays that showed similar results, each performed with n = 3 technical replicates. *P < 0.05 compared with the untreated control. Data represent mean ± SEM. (D) Transwell migration assay of Ca9/K17+ (C-4) treated with RPM and/or PRF (left panel). Transwell invasion assay of Ca9/K17+ (C-4) treated with RPM and/or PRF (right panel). Representative graphs of three assays that showed similar results, each performed with n = 3 technical replicates. **P < 0.01 and *P < 0.05 compared with the untreated control. Data represent mean ± SEM.

To explore the mechanisms of the KRT17-mediated stimulation of proliferation and migration, we examined the signaling molecules that are important in wound healing. Western blot analysis revealed that the expression levels of phosphorylated AKT1 (pAKT1), MTOR, and phosphorylated EIF4EBP1 (eukaryotic translation initiation factor 4E binding protein 1) increased in Ca9/K17+ compared with Ca9 (Fig 4A). The expression in each clone was measured by densitometry, normalized against TUBB and then against the parental control cells. The densitometric analysis showed that the expression levels of pAKT1, MTOR and pEIF4EBP1 increased in all the Ca9/K17+ clones (Fig 4B) compared with the control. In contrast, the expression levels of AKT1, pAKT1, MTOR and pEIF4EBP1 decreased in all the HSC3/K17- clones compared with the control (Fig 4B). The results indicate that KRT17 stimulates the Akt/mTOR signaling pathway. Next, we examined the effect of MTOR inhibitor rapamycin and AKT1 inhibitor perifosine on the proliferation and migration of Ca9/K17+. Rapamycin significantly inhibited proliferation at concentrations greater than 0.1 μM (P < 0.05), whereas perifosine did not (P > 0.1; Fig 4C), suggesting that the mTOR pathway contributed to the KRT17-stimulated increase in proliferation, whereas AKT1 had a limited role in proliferation. In the transwell migration and invasion assays, both rapamycin and perifosine inhibited migration and invasion (P < 0.05; Fig 4D). Combined treatment with rapamycin and perifosine inhibited migration and invasion in an additive manner (P < 0.01; Fig 4D), suggesting that the KRT17-stimulated migration was via activation of the Akt/mTOR signaling pathway.

### KRT17 promotes SLC2A1 expression and glucose uptake

**Fig 5:**
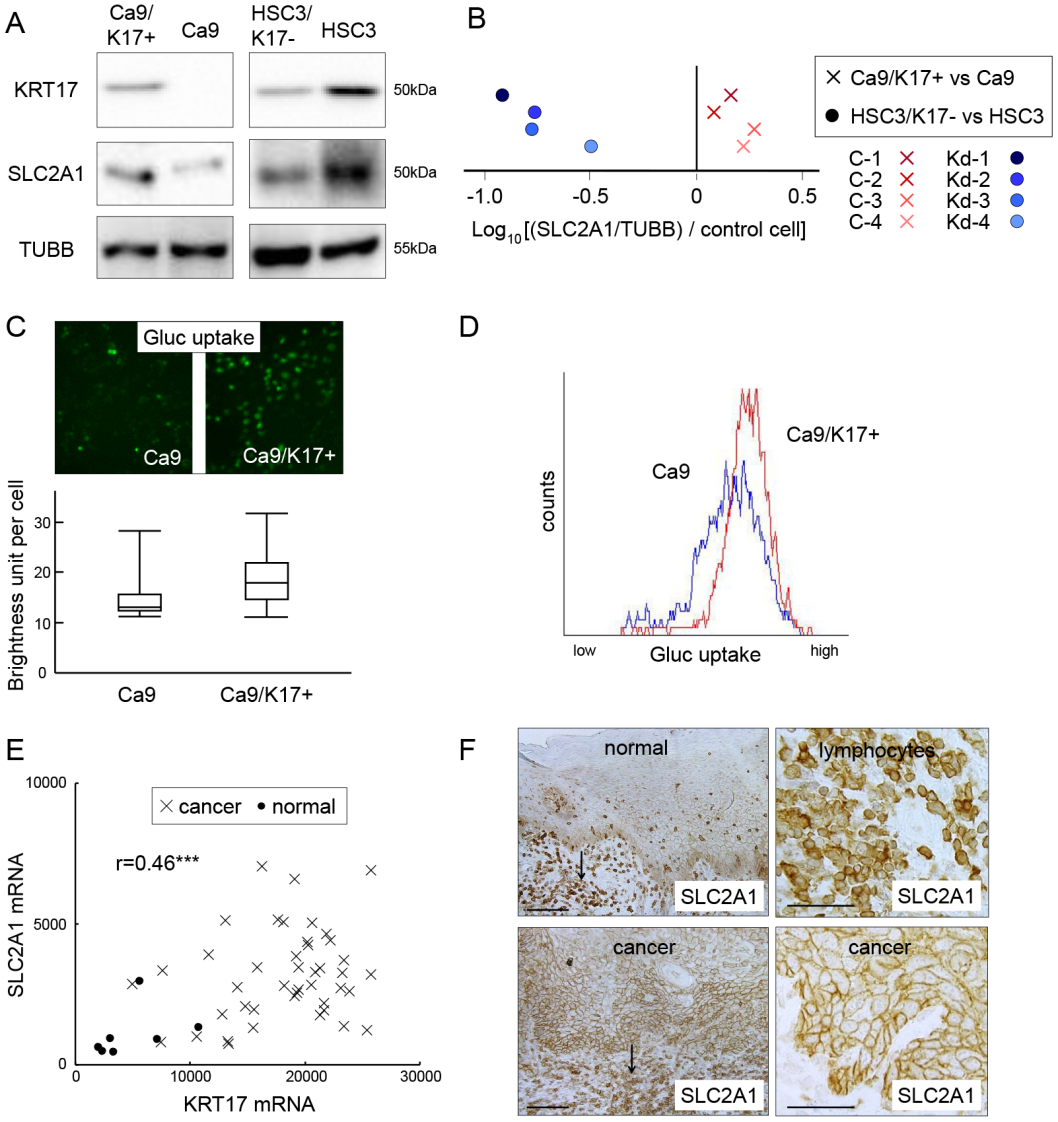
KRT17 upregulates SLC2A1 and glucose uptake. (A) Representative images of western blot analysis of Ca9/K17+ (C-4), Ca9, HSC3/K17- (Kd-4) and HSC3 for detecting KRT17, SLC2A1, and beta1-tubulin (TUBB). Representative images of three assays. (B) Expression of SLC2A1 in Ca9/K17+ (C-1, C-2, C-3, and C-4) and HSC3/K17- (Kd-1, Kd-2, Kd-3, and Kd-4) compared with Ca9 and HSC3, respectively, as revealed by densitometric analysis of the western blots. Expression level of protein X in a clone was normalized against TUBB and then against the control cells using the formula (Value of protein X in the clone / Value of TUBB in the clone) / (Value of protein X in the control cells / Value of TUBB in the control cells). The normalized expression levels were plotted on a log scale. Representative results of three independent assays. (C) Glucose uptake assay of Ca9 and Ca9/K17+ (C-4) cells as measured by fluorescence microscopy. The cells were treated with a fluorescent glucose analogue for 2 h. The fluorescence image was digitally analyzed and the signal in individual cells was depicted as a brightness unit. The box plot illustrates the maximum, third quartile, median, first quartile and minimum signals of 100 cells. Representative results of three assays. (D) Glucose uptake assay as measured by flow cytometry. The cells were treated with the fluorescent glucose analogue for 2 h and were harvested for flow cytometric analysis (n = 1000). (E) cDNA microarray analysis of *KRT17* and *SLC2A1* in 43 oral squamous cell carcinomas (OSCCs) and 7 normal controls. *SLC2A1* was significantly upregulated in OSCC (P < 0.001), with an approximate three-fold increase both in the mean and the median value. There was a positive correlation between *SLC2A1* and *KRT17* expression (r = 0.46; ***P < 0.001). The scales on the horizontal and vertical axes represent absolute signal values. Crosses denote each OSCC case and filled circles denote normal controls. (E) Immunohistochemical expression of SLC2A1 in normal tongue epithelium and tongue cancer. Expression was greatest in lymphocytes (arrows in left upper and lower panels). In the normal oral epithelium, SLC2A1 was weakly expressed in the basal and spinous cells (left upper panel). In OSCC, SLC2A1 was upregulated, showing a level of expression comparable with lymphocytes (left and right lower panels). Scale bar, 100 μm.

Next we examined the relationship between KRT17 and SLC2A1. Western blot analysis revealed that the expression of SLC2A1 increased in Ca9/K17+ compared with Ca9, and decreased in HSC3/K17- compared with HSC3 (Fig 5A). Densitometric analysis showed an increase in SLC2A1 expression in all the Ca9/K17+ clones and a decrease of SLC2A1 expression in all the HSC3/K17- clones compared with the controls (Fig 5B). We measured glucose uptake using the fluorescently-labeled deoxyglucose analog 2-NBDG. In Ca9/K17+, an increase of glucose uptake was observed by microscopic examination (Fig 5C, upper panel). The fluorescence signals in each cell (n = 100) were measured by densitometry, which revealed a significant increase of glucose uptake in Ca9/K17+ compared with Ca9 (P < 0.01; Fig 5C, lower panel). Flow cytometry confirmed the increase of glucose uptake in Ca9/K17+ cells (n = 1000; P < 0.05; Fig 5D). The cDNA microarray analysis demonstrated that the expression of *SLC2A1* was significantly upregulated (2.92-fold; P < 0.01) in OSCC compared with the normal epithelium (Fig 5E), and there was a positive correlation between *SLC2A1* and *KRT17* (r = 0.46; P < 0.001). SLC2A1 expression was observed in the normal oral epithelium at a lower level than in lymphocytes, as revealed by immunohistological analysis (Fig 5F, upper panels). In OSCC, SLC2A1 was markedly upregulated to a level comparable with that in the lymphocytes (Fig 5F, lower panels). These results indicate that KRT17 stimulates glucose uptake by increasing the expression of SLC2A1.

### Concurrent upregulation of pAKT1, MTOR, and SLC2A1 with KRT17 in OSCC

**Fig 6:**
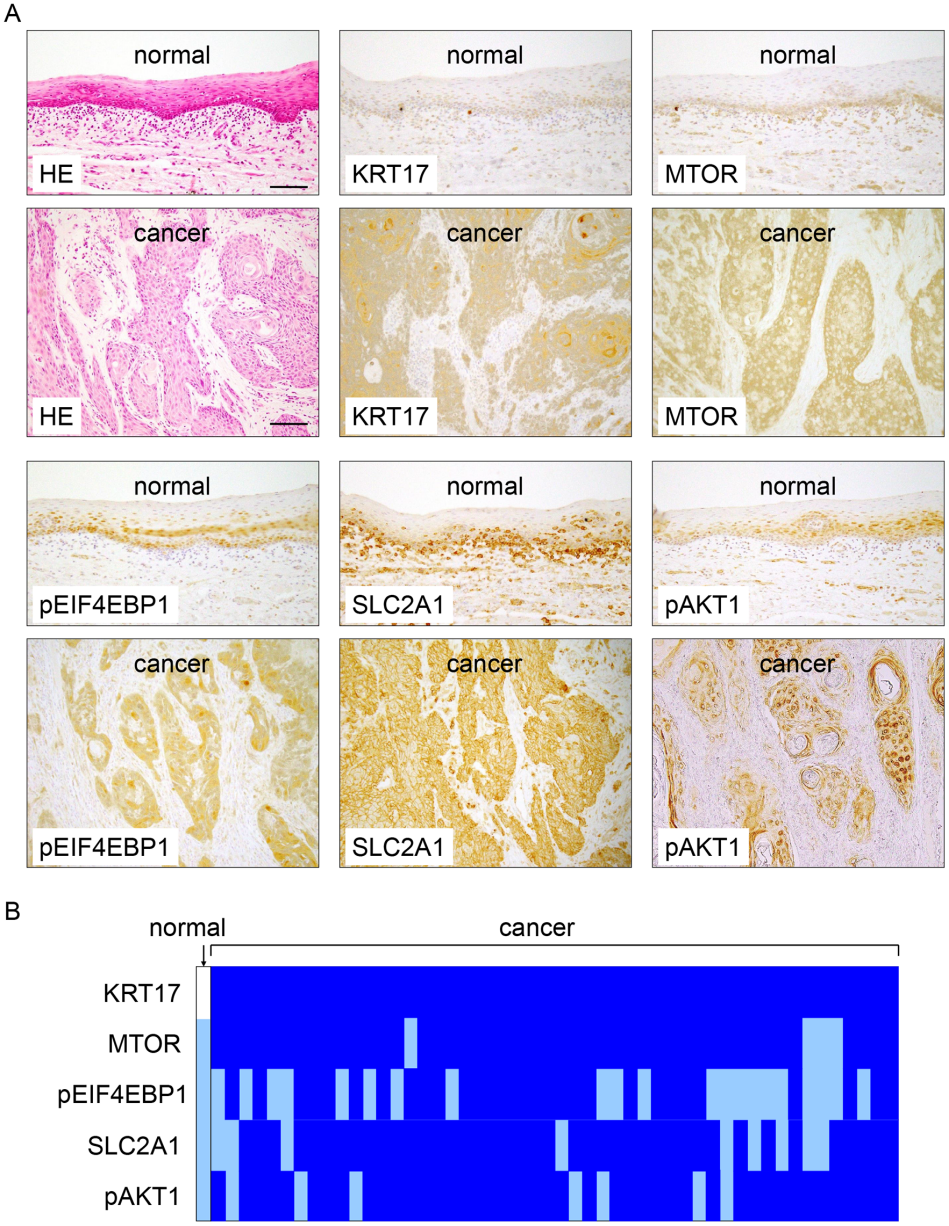
KRT17, pAKT1, MTOR, phosphorylated EIF4EBP1 (pEIF4EBP1) and SLC2A1 were concurrently upregulated in oral squamous cell carcinoma (OSCC). (A) Representative images of immunohistochemical expression of KRT17, MTOR, pEIF4EBP1, SLC2A1 and pAKT1 in normal oral epithelium (normal) and OSCC (cancer). HE, hematoxylin and eosin staining. Scale bar, 100 μm. (B) Schematic representation of the immunohistochemical expression of KRT17, MTOR, pEIF4EBP1, SLC2A1 and pAKT1 in 50 OSCC cases. A blue square denotes increased expression in the individual OSCC case compared with the normal epithelium as an internal control. A white square denotes no immunohistochemical expression. A light-blue square denotes weak staining comparable to that in the normal epithelium. There was not a single case of downregulation of these proteins in OSCC.

To confirm the positive correlations with KRT17, we immunohistochemically investigated the expression levels of pAKT1, MTOR, pEIF4EBP1 and SLC2A1 in another 50 OSCC cases that were different from those in Fig 1. The induction of KRT17 in cancer was confirmed in all the cases. pAKT1, MTOR, pEIF4EBP1 and SLC2A1 were conspicuously upregulated in OSCC compared with the normal epithelium (Fig 6A). Semiquantitative evaluation revealed that the elevated expression of pAKT1, MTOR, pEIF4EBP1 and SLC2A1 in cancer was evident in 47 (94%), 30 (60%), 43 (86%) and 42 (84%) cases, respectively (Fig 6B). These observations support the results of cell culture experiments showing that KRT17 stimulated the Akt/mTOR pathway and SLC2A1 expression.

### KRT17 knockout inhibits tumor growth *in vivo*

**Fig 7:**
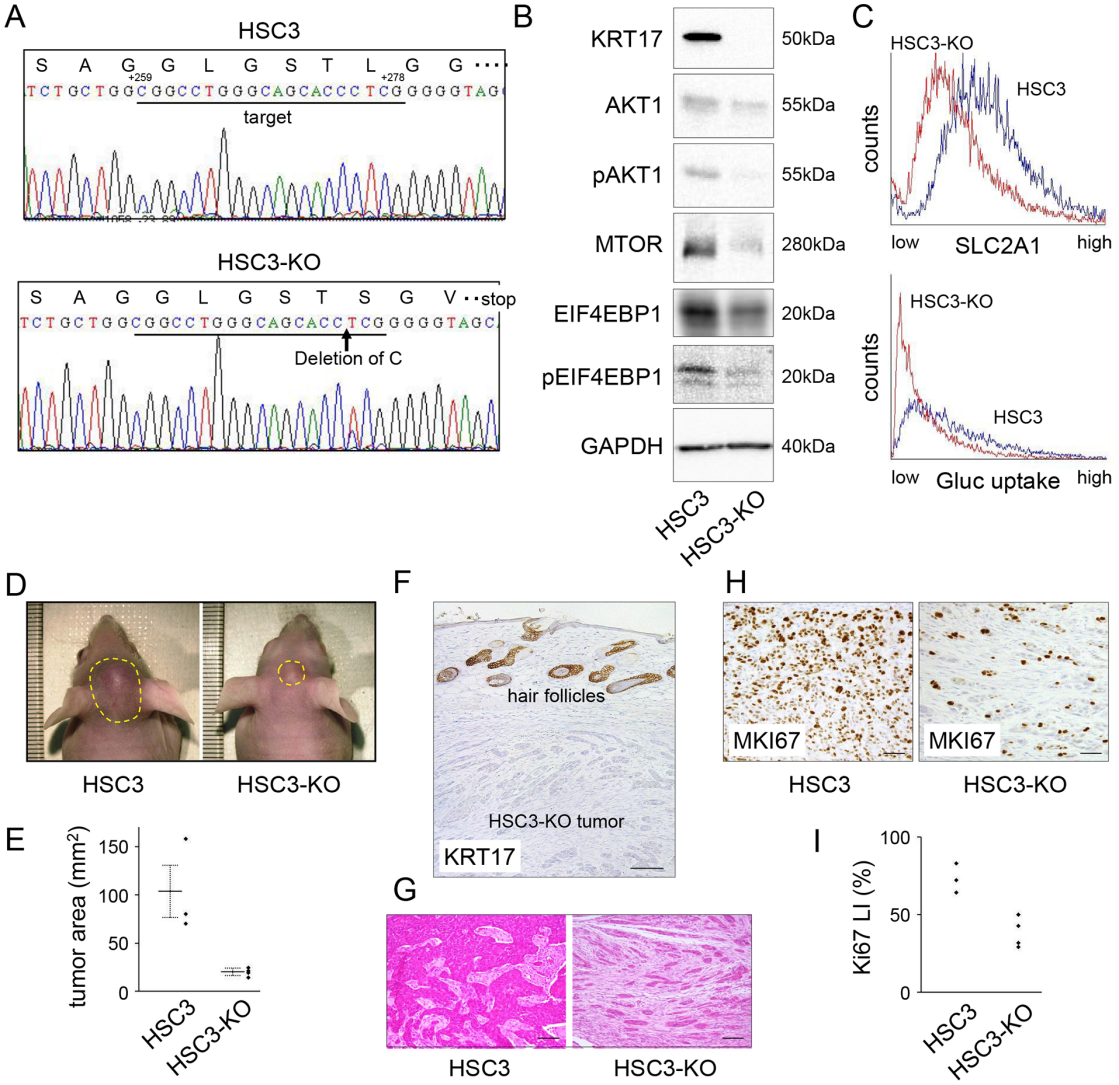
KRT17 knockout inhibited tumor growth. (A) Confirmation of *KRT17* mutation in the KRT17-knockout cells (HSC3-KO). HSC3 was transfected with *pSpCas9(BB)-2A-GFP* carrying the *KRT17* target sequence in *KRT17* exon 3 and cloned by limited dilution following fluorescence activated cell sorting of transfected cells. Six independent clones, in which KRT17 protein expression was absent, were established. Of those, one clone (HSC3-KO) exhibited homo-allelic single-base deletion as revealed by PCR and direct sequencing of the genomic DNA, resulting in a frame shift and a premature stop codon. +259 and +278 denote the nucleotide positions corresponding to *KRT17* cDNA when the A of the ATG of the initiator methionine codon is designated as position +1. (B) Western blot analysis of HSC3 and HSC3-KO, demonstrating the absence of KRT17 and reduced expression of phosphorylated pAKT1, MTOR, and pEIF4EBP1 in HSC3-KO. (C) Reduced SLC2A1 expression and glucose uptake in HSC3-KO, as revealed by flow cytometry. (D) Cells (5 x 10^5^) of HSC3 or HSC-KO were subcutaneously injected into the cephalic skin of nude mice (n = 4). One mouse transplanted with HSC3 died of an unknown cause on day 10. HSC3-KO cells developed smaller tumors than HSC3. The photographs were taken on day 15. The tumor areas are encircled by yellow dashed lines. (E) The tumor area was calculated following elliptic substitution of the macroscopic tumor margin using the photograph of the vertical view. The bold lines depict mean tumor areas. The error bars represent standard errors. (F) Immunohistochemical examination of the HSC3-KO tumor developed in the nude mice, confirming negative expression of KRT17. Since the antibody recognizes both human and mouse KRT17, the physiological expression of KRT17 was observed in the hair follicles. Scale bar, 200 μm. (G) Histology of the HSC3 tumor and the HSC3-KO tumor. The HSC3 tumor was composed of medium-to-large-sized tumor nests, whereas the HSC3-KO tumor was composed of small islands. Scale bar, 200 μm. (H) Immunohistochemical expression of MKI67 in the HSC3 tumor and the HSC3-KO tumor. Note that there were fewer cancer cells in the HSC3-KO photograph than in the HSC3 photograph. Scale bar, 200 μm. (I) The Ki-67 labeling indices (LI) were calculated as the percentage of MKI67-positive nuclei in the cancer cells after counting at least 1,000 tumor cells at X200 magnification. The tumor areas that were closest to the epidermis were used for analysis.

To examine the behavior of cancer cells in tissue, we subcutaneously injected Ca9/K17+, Ca9, HSC3/K17- and HSC3 into the cephalic dermis of athymic mice. The transplantability of Ca9 was low and tumor formation was not apparent in either Ca9 or Ca9/K17+. HSC3/K17- and HSC3 formed tumors; however, immunohistochemical staining unexpectedly showed that KRT17 expression had been restored in HSC3/K17- (data not shown). We speculated that the knockdown effect had been attenuated because of an absence of antibiotic selection. To overcome this problem, we created *KRT17* knockout cells using CRISPR/Cas9 genome editing (Fig 7A, referred to as HSC3-KO). Western blot analysis revealed a total absence of KRT17 (Fig 7B). HSC3-KO showed reduced expression of AKT1, pAKT1, MTOR, and pEIF4EBP1 compared with HSC3 (Fig 7B). SLC2A1 expression and glucose uptake decreased in HSC3-KO compared with HSC3 (Fig 7C). In the tumor xenograft experiments, tumors were recognized on gross examination 5 days after injection of HSC3 and 7 days after injection of HSC3-KO, on average. Fifteen days after injection, the tumors formed by HSC3-KO (n = 4) were significantly smaller than those formed by HSC3 (n = 3, one mouse died as a result of an unknown cause on day 10) (P < 0.05; Fig 7D and 7E). The HSC3-KO tumors were confirmed to be negative for KRT17 by immunohistochemistry (Fig 7F). The tumor histology showed a considerable difference between HSC3 and HSC3-KO; the HSC3 tumor was composed of medium-to-large-sized tumor nests, whereas the HSC3-KO tumor was mainly composed of small tumor islands (Fig 7G). Immunohistochemical examination of MKI67 (marker of proliferation, Ki67) revealed that MKI67-positive tumor cells were less common in the HSC3-KO tumor compared with the HSC3 tumor (Fig 7H). To balance the differences of tumor microenvironments, MKI67-positive tumor cells were counted at the tumor area closest to the epidermis, which was about 0.5 mm below the surface, and the Ki67 labeling index was calculated. The HSC3-KO tumors showed lower Ki-67 labeling indices than the HSC3 tumors (Fig 7I). Collectively, these results indicate that KRT17 supports tumor growth *in vivo*.

### The relationship between KRT17 and SFN is not evident in the cancer cells

**Fig 8:**
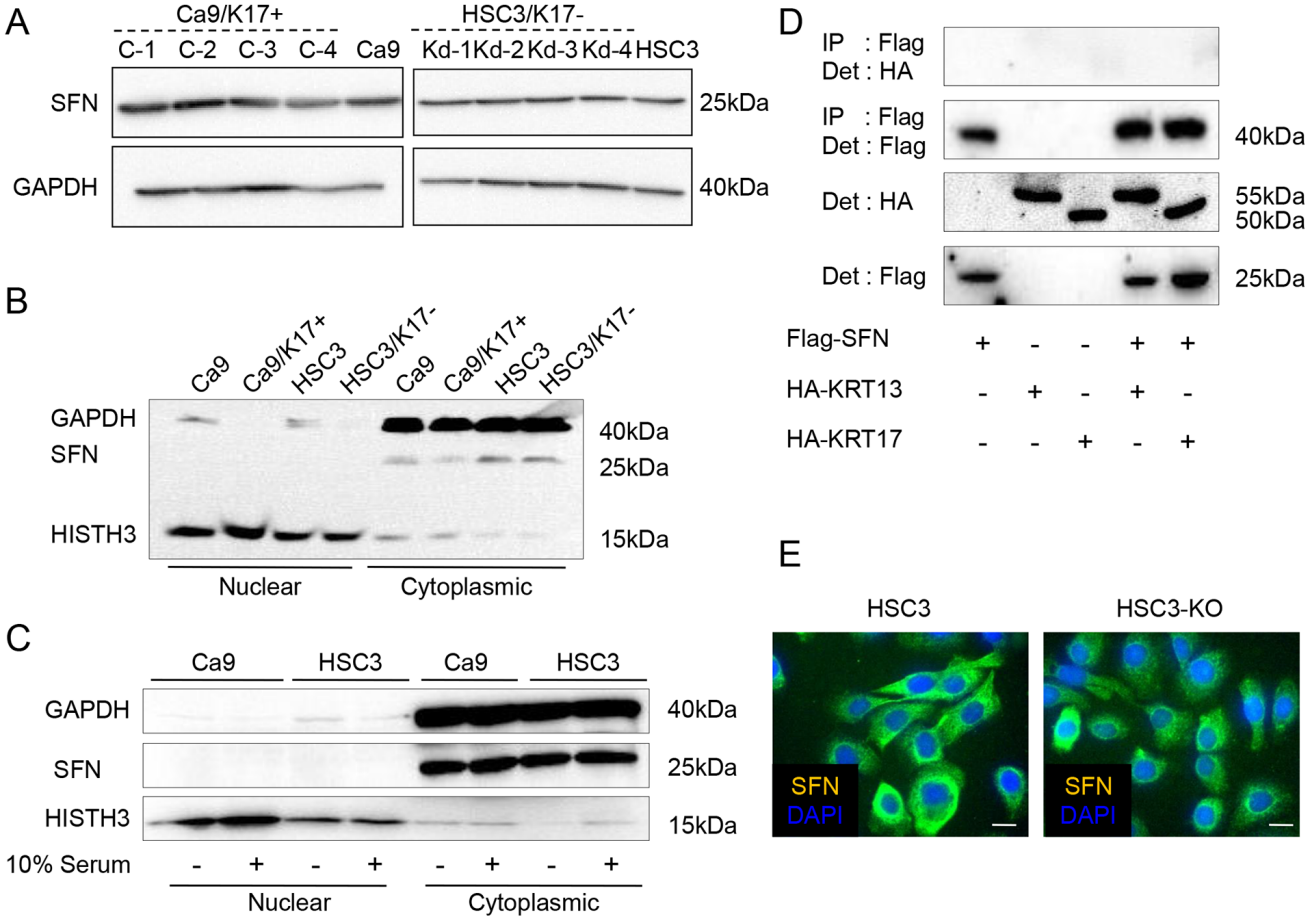
No direct relationship between KRT17 and stratifin (SFN). (A) Western blot analysis of Ca9/17+ (C-1, C-2, C-3, and C-4), Ca9, HSC3/K17- (Kd-1, Kd-2, Kd-3, and Kd-4) and HSC3 for detecting SFN and GAPDH. (B) SFN was detected only in the cytoplasmic fractions of the cell lysates. Ca9, Ca9/K17+ (C-4), HSC3 and HSC3/K17- (Kd-4) were lysed and cytoplasmic and nuclear protein fractions were separately extracted. Western blot analysis for detecting SFN, GAPDH and HISTH3. Nuclear translocation or change in the expression level of SFN was not observed. Representative graphs of two assays that showed similar results. (C) Serum starvation did not alter the cytoplasmic localization of SFN. After serum starvation for 12 h, Ca9 and HSC3 cells were lysed and subjected to SDS-PAGE followed by western blot analysis. (D) Absence of KRT13 or KRT17 coprecipitation in SFN immunoprecipitate. 293T cell were transfected by the indicated combinations of plasmids and immunoprecipitation was performed using anti-Flag antibody followed by western blot analysis using an anti-HA antibody. (E) Cytoplasmic localization of SFN in HSC3 and HSC3-KO. Immunofluorescent images showing cytoplasmic staining of SFN (green) counterstained with DAPI (blue).

We determined whether KRT17 works by modulating the nucleocytoplasmic localization of SFN in cancer cells, as has previously been observed in mouse epidermal cells [12]. SFN expression levels in Ca9/K17+ and HSC3/K17- were the same as in the controls (Fig 8A). SFN was detected only in the cytoplasmic fraction with no significant difference between the KRT17-engineered cells and the controls (Fig 8B). Serum-starvation did not alter the cytoplasmic localization of SFN in these cells (Fig 8C), or in the HFS cells (data not shown). The effects of KRT17 in Ca9/K17+ and HSC3 seemed independent of SFN. Next, we investigated the interaction between SFN and KRT17 by immunoprecipitation. Because we wanted to use a well-characterized antibody for immunoprecipitation and detection, we prepared plasmids carrying *Flag*-tagged *SFN* and *HA*-tagged *KRT17*. *HA*-tagged *KRT13* was also prepared to determine if the association was specific for KRT17. Ca9 and HSC3 were co-transfected with these plasmids, but we could not detect the coprecipitation of SFN and KRT17 (data not shown). The efficiencies of transient transfection to HSC3 and Ca9 were low (less than 10%); this result may have been due to insufficient amounts of protein. Therefore, we then used 293T cells to induce robust expression. There was no endogenous expression of KRT13 and KRT17 in 293T (data not shown). Immunoprecipitation of SFN was confirmed in 293T cells, but coprecipitation of KRT17 or KRT13 was not detected (Fig 8D). Immunocytochemistry showed that SFN localized in the cytoplasm in both HSC3 and HSC3-KO. Nuclear expression was not evident (Fig 8E). In brief, we did not obtain evidence for a relationship between KRT17 and SFN in the human cells.

## Discussion

Our previous studies on the keratins that are downregulated in OSCC have uncovered some essential characteristics of OSCC. The loss of KRT4 and KRT13 expression represents maturation abnormality in the neoplastic epithelium [11]. KRT15 and KRT19 are also downregulated in OSCC, and the expression levels of the remaining KRT15 and KRT19 represent differentiation and the invasive potential of the cancer, respectively [19]. The loss of these physiological keratins appears to be compensated by ectopic induction of other keratins. This is not random upregulation of diverse keratin subtypes; instead, specific keratins are markedly induced [11]. In the present study, we demonstrated that the induction of KRT17 is a fundamental feature of OSCC. KRT17 showed the same cellular localization as the other keratins in cancer cells, indicating that KRT17 was correctly incorporated into the cytoskeleton. This suggests that KRT17 replaces the physiological keratins and plays a substantial role in a controlled manner even in OSCC.

The existence of subtype-specific functions is still controversial, but mounting evidence indicates that keratins regulate diverse physiological processes in a context dependent manner [4, 25]. The unique expression pattern of KRT17 during development appears to reflect its specific function. KRT17 expression is initiated in the early stages of skin development at the epithelial placodes [7]; it is maintained during epithelial appendage formation and continues in basal/myoepithelial cells [3]. Despite the differences in final forms, the processes of appendage formation share the common cellular dynamics of proliferation, migration, invagination, and branching, suggesting that KRT17 may play a role in these developmental dynamics [7]. Cancer growth and spread are also propelled by these morphogenetic cell dynamics. Indeed, we found that KRT17 promoted tumor growth by stimulating the Akt/mTOR pathway and glucose uptake. This signaling cascade also plays a crucial role in wound healing [26, 27]. Therefore, both in physiological and pathological contexts, KRT17 appears to fulfill the role of a so-called signaling platform [28]: an emerging concept which may help explain the diverse impact of keratin functions. However, the mechanisms by which KRT17 acts as the signaling platform remain elusive.

Data obtained from the keratin-null mice suggest that keratins modulate the membranous localization of SLC2A1, and that the level of surface SLC2A1 expression regulates mTOR signaling in an adenosine monophosphate-activated protein kinase (AMPK)-dependent manner [14]. This theory places SLC2A1 upstream of MTOR. However, the elevated expression of SLC2A1 may also be the consequence of activation of the Akt/mTOR pathway, considering the previous studies showing that MTOR regulates the insulin-dependent expression of SLC2A1 [29, 30] and promotes trafficking of SLC2A1 to the cell membrane [31], also that AKT1 stimulates the transcription [32] and the cell surface localization [33] of SLC2A1. Feedback mechanisms orchestrate these molecules, thereby maintaining proper glucose uptake [34], but much more research is required to understand how KRT17 participates in the regulation of glucose metabolism.

In normal mouse cutaneous keratinocytes, KRT17 mediates the effect of SFN on the Akt/mTOR pathway by retaining SFN in the cytoplasm [12]. Mikami et al. reported nuclear translocation of SFN following KRT17 knockdown in the OSCC cell line ZK-1, as revealed by immunocytochemistry [35]. However, such a relationship between KRT17 and SFN was not evident in the human cells examined in this study, suggesting that KRT17 also activates the Akt/mTOR pathway by other means in OSCC.

Keratins modulate the tumor necrosis factor (TNF) signaling pathways through physical interaction with the signaling proteins. For example, KRT8 and KRT18 associate with TNFRSF1B (tumor necrosis factor receptor superfamily, member 1B/TNFR2) and attenuate its signal transduction [36], and KRT17 interacts with TRADD (TNFRSF1A-associated via death domain) and regulates TNF alpha signaling [37]. The interaction between keratins and signaling proteins is an interesting aspect of keratin behavior.

The expression of KRT17 is controlled by the transcription factor GLI2 [38, 39], which acts as an oncogene in OSCC [40]. *Krt5-Gli2* transgenic mice develop multiple basal cell carcinomas of the skin that are characterized by robust expression of KRT17 in the tumor cells [41]. Genetic ablation of *Krt17* delays tumor development in transgenic mice [42], suggesting that GLI2-driven KRT17 expression has tumor-promoting activity. In humans, GLI2 is detected in about half of OSCC cases and is significantly associated with poor clinical outcomes [43]. These findings suggest that the GLI2-KRT17 axis may play an important role in the progression of OSCC. KRT17 also modulates the expression of the transcriptional regulator *AIRE* (autoimmune regulator) in diseased epithelia, which induces the expression of proinflammatory cytokines and supports tumor progression [44]. Collectively, these findings, including the present ones, highlight the importance of KRT17 as a multifunctional promoter of tumorigenesis.

Our study reinforces the concept that the cellular properties of cancer are regulated by a series of molecules similar to those found in wound healing. KRT17, which is an inductive keratin in the regenerative epithelium, acts in OSCC as a pathogenic keratin that facilitates tumor growth through stimulation of multiple signaling pathways.

## Supporting Information

S1 Fig Absence of KRT17 expression in the normal oral epithelium. HE, hematoxylin and eosin stain. Scale bar, 100 μm.
(TIF)

S2 Fig Upregulation of KRT5, KRT6 and KRT17 in OSCC. (A) Representative immunohistochemistry images of OSCC, showing the expression of KRT5, KRT6 and KRT17 in cancer. (B) Confirmation of the immunohistochemistry results in representative 3 cases (#1, #2, and #3) by western blot analysis. Normal epithelium (N) and OSCC (C) were separately macrodissected from formalin-fixed paraffin-embedded tissue specimens and proteins were extracted.
(TIF)

S3 Fig Colocalization of KRT5, KRT6 and KRT17 in HSC3 cells. Immunocytochemistry. Scale bar, 10 μm.
(TIF)

S4 Fig KRT17 expression in primary human foreskin cells. Western blot analysis.
(TIF)

S5 Fig KRT5, KRT6, KRT16 and KRT17 expression in various oral cancer cell lines. Western blot analysis.
(TIF)

S6 Fig KRT5, KRT6 and KRT17 expression in Ca9/K17+ cells. Immunocytochemistry. Scale bar, 10 μm.
(TIF)
