# Supplementary figures and images for: Keratin 17 Is Induced in Oral Cancer and Facilitates Tumor Growth
Source: PLoS One. 2016 Aug 11;11(8):e0161163. doi: 10.1371/journal.pone.0161163 (PMC4981360; doi:10.1371/journal.pone.0161163)

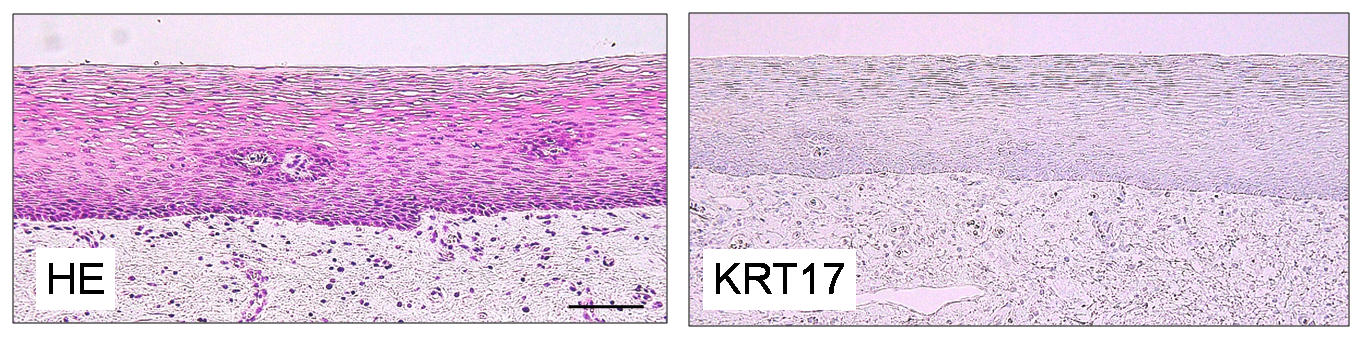

Supplement: S1 Fig — HE, hematoxylin and eosin stain. Scale bar, 100 μm. (TIF) [file pone.0161163.s001.tif]

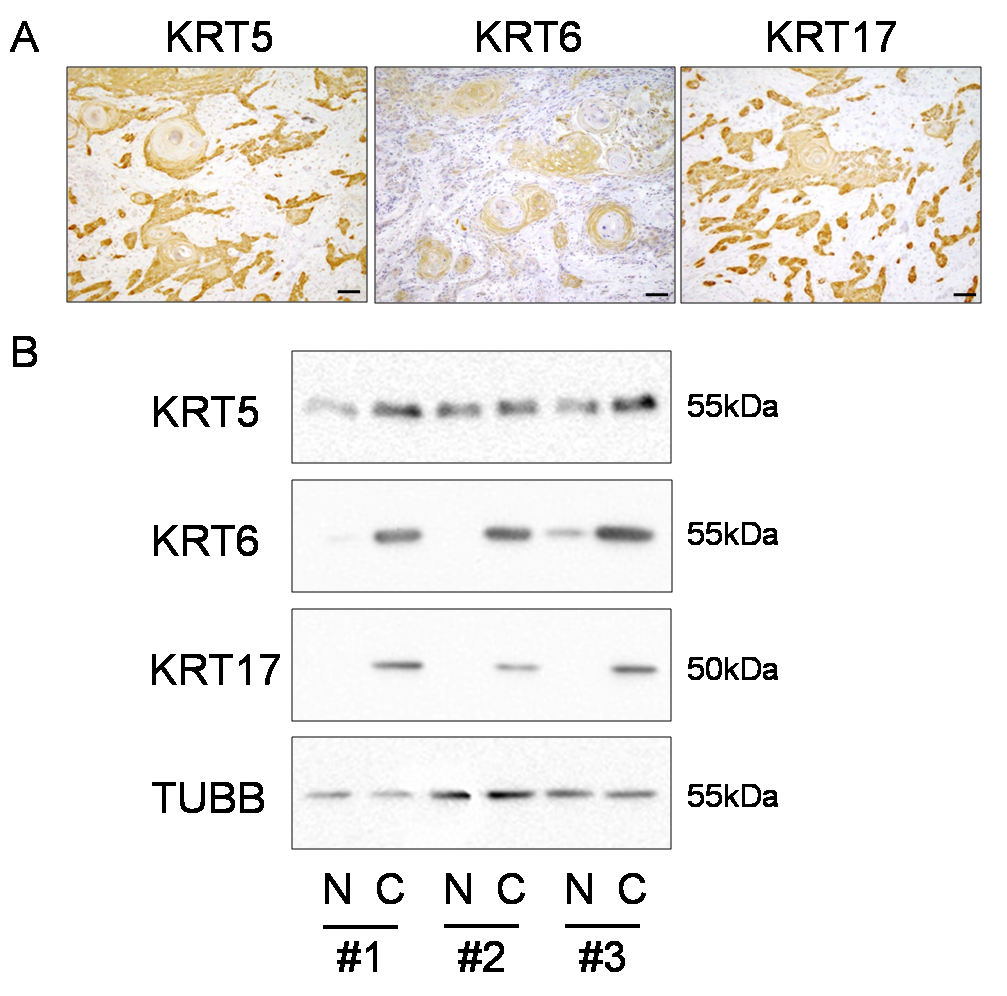

Supplement: S2 Fig — (A) Representative immunohistochemistry images of OSCC, showing the expression of KRT5, KRT6 and KRT17 in cancer. (B) Confirmation of the immunohistochemistry results in representative 3 cases (#1, #2, and #3) by western blot analysis. Normal epithelium (N) and OSCC (C) were separately macrodissected from formalin-fixed paraffin-embedded tissue specimens and proteins were extracted. (TIF) [file pone.0161163.s002.tif]

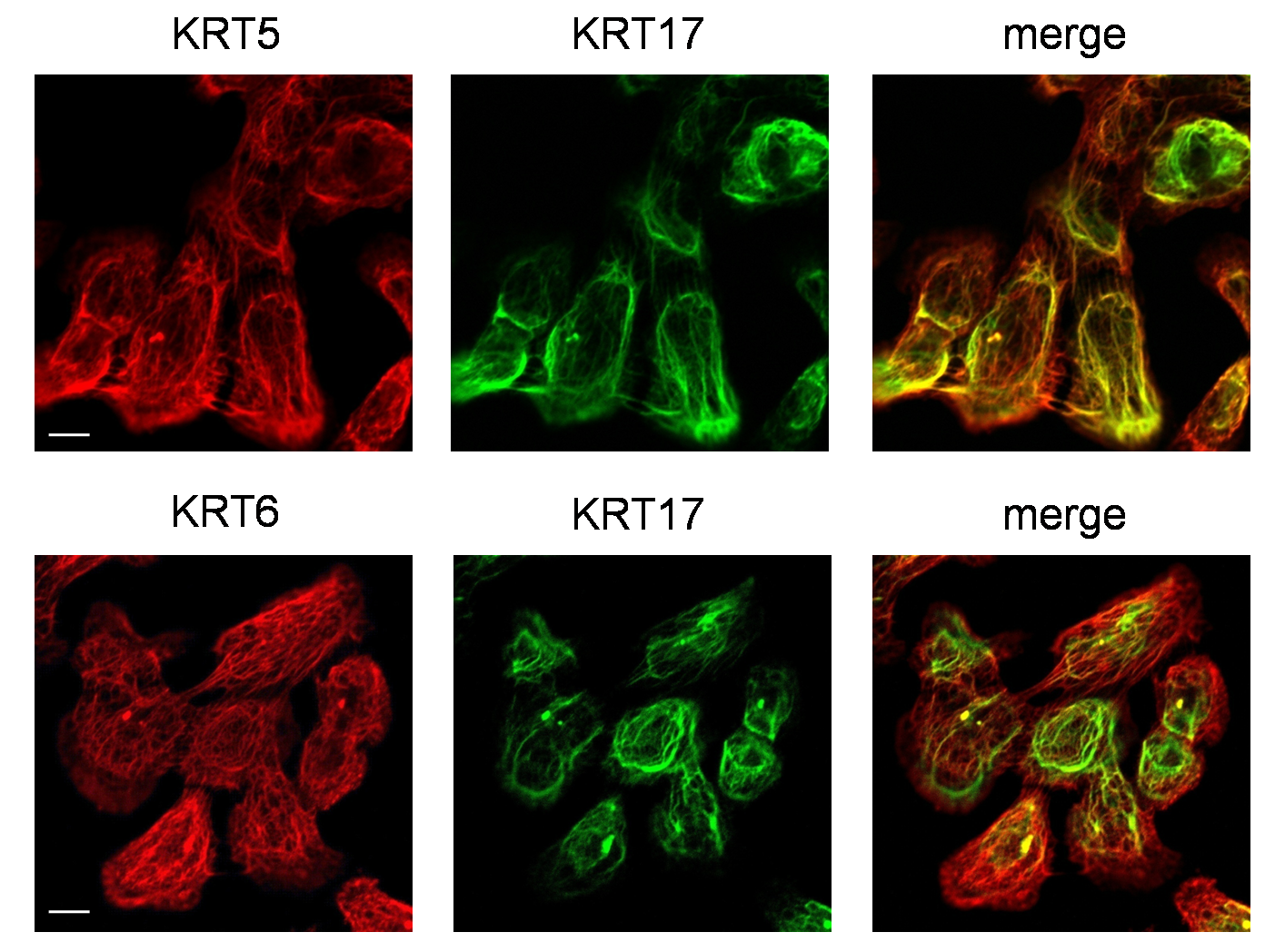

Supplement: S3 Fig — Immunocytochemistry. Scale bar, 10 μm. (TIF) [file pone.0161163.s003.tif]

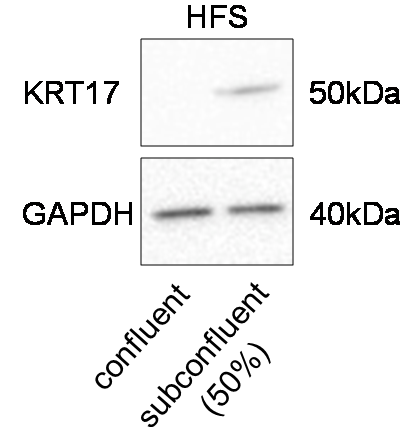

Supplement: S4 Fig — Western blot analysis. (TIF) [file pone.0161163.s004.tif]

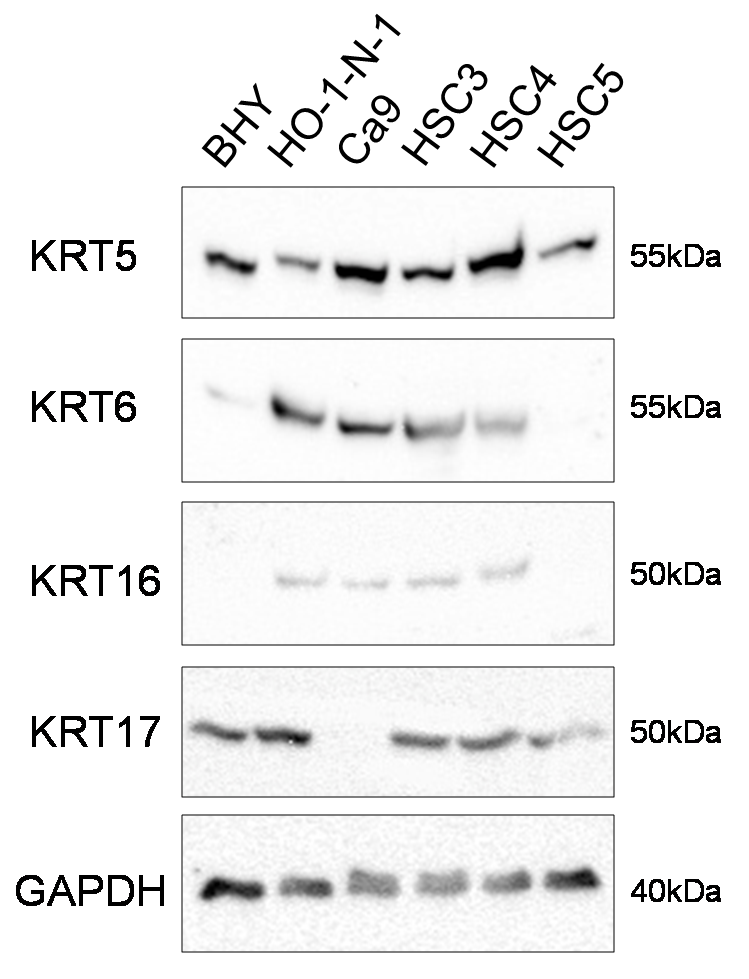

Supplement: S5 Fig — Western blot analysis. (TIF) [file pone.0161163.s005.tif]

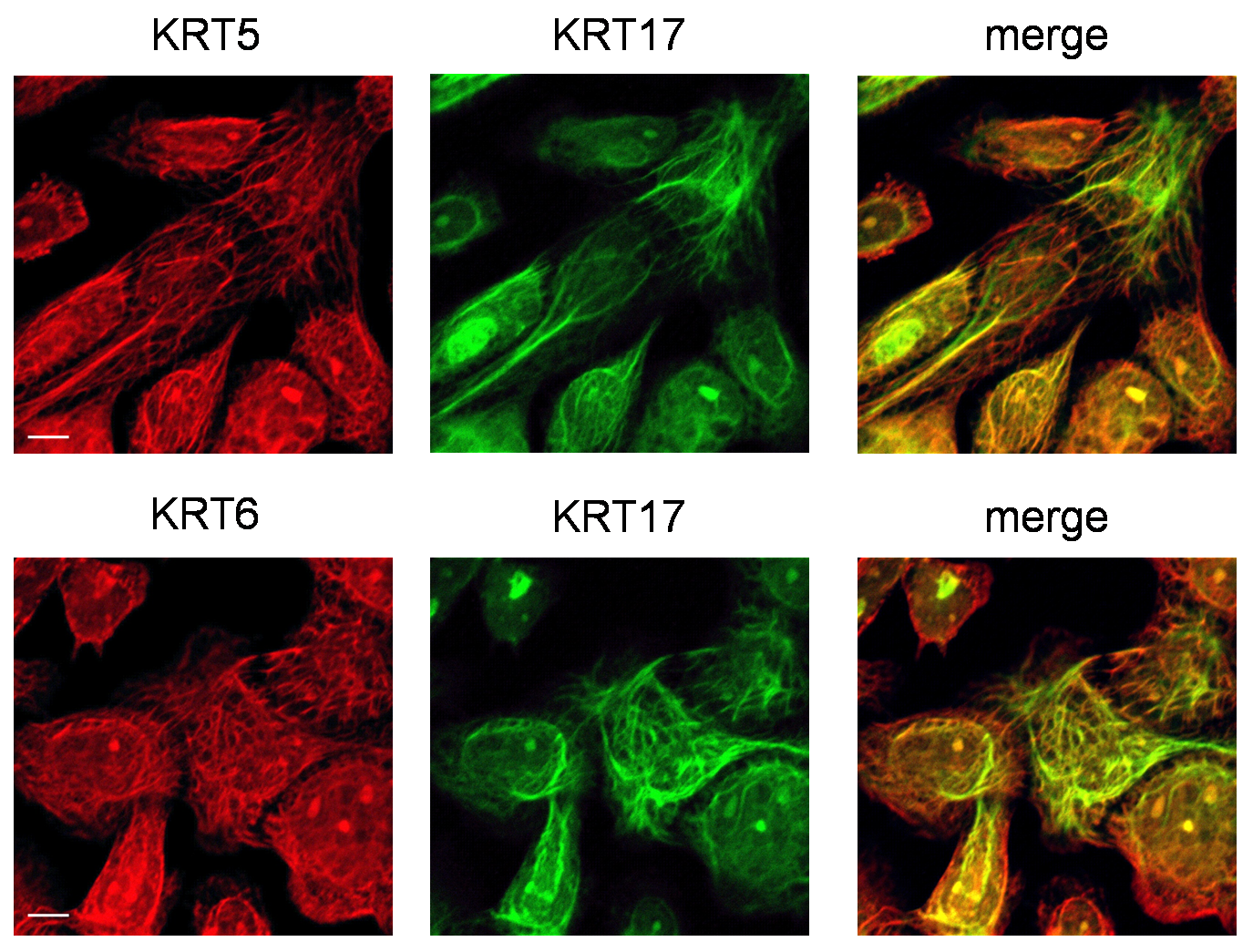

Supplement: S6 Fig — Immunocytochemistry. Scale bar, 10 μm. (TIF) [file pone.0161163.s006.tif]
